# Meibomian gland dysfunction patients benefit in ocular parameters and tear chemokines after thermal pulsation treatment

**DOI:** 10.7150/ijms.76603

**Published:** 2023-01-01

**Authors:** Wenting Liu, Tong Lin, Lan Gong

**Affiliations:** 1 Department of Ophthalmology and Vision Science, The Eye, Ear, Nose and Throat Hospital of Fudan University, Shanghai 200031, P.R. China; 2 NHC Key Laboratory of Myopia, Laboratory of Myopia, Chinese Academy of Medical Sciences, Fudan University, Shanghai 200031, P.R. China; 3 Department of Ophthalmology, Huadong Hospital of Fudan University, Shanghai, China

**Keywords:** meibomian gland dysfunction, thermal pulsation treatment, tear chemokines, CXCL cytokines, tear film stability

## Abstract

**Objectives:** To investigate the effect of thermal pulsation treatment on meibomian gland function, ocular parameters and tear inflammatory cytokines compared with the warm compress group.

**Methods:** Twenty-five participants with MGD underwent a 12-minute thermal pulsation treatment, while 25 participants with MGD underwent manual warm compress treatment. MGD related parameters, including meibomian gland function (MGE, MQ and lid margin), tear stability (NIKBUT, FBUT and LLT), tear secretion (SIT, and TMH), were examined and OSDI questionnaire was also obtained. Tear chemokines (MIG, IFN-γ, IL-8, IP-10 and MCP-1) were examined and analyzed the correlations with MGD related parameters and OSDI.

**Results:** Compared with warm compress subjects, OSDI, lid margin and tear stability were found improved more in thermal pulsation treatment at 3 months (OSDI: *p = 0.014, lid margin: *p = 0.021, LLT: **p = 0.008, CFS: *p = 0.028). The level of IP-10 and MIG decreased more in thermal pulsation group than in warm compress group (IP-10: *p = 0.021, MIG: *p = 0.039). IP-10 was positively correlated with MQ (r = 0.522, *p = 0.037) and negatively correlated with tear stability (r = -0.613, **p = 0.002), and OSDI was only positively correlated with IL-8 (r = 0.679, ***p < 0.001). The decrease of MIG was positively correlated with less corneal epithelium injury (r = 0.557, **p = 0.006) and meibograde (r = 0.49, *p = 0.019).

**Conclusions:** Thermal pulsation treatment obviously improved MGD probably by attenuating tear CXCL chemokines in ocular surface of MGD patients, which demonstrated an efficacy and well-tolerated therapy in clinical.

## Introduction

Meibomian gland dysfunction (MGD), a chronic abnormality of the meibomian glands, remarkably affects tear film stability and lead to various ocular surface disease problems 1. In ophthalmology clinics, MGD is one of the most common disorders routinely, so it can be considered as a public health problem 2. The global prevalence of MGD was reported to range from 10-20%, while the prevalence of MGD in the Eastern Asian even achieved more than 50% 3. Considering that the core characteristic of MGD is terminal meibomian gland duct obstruction accompanied with or without qualitative/quantitative changes in the glandular secretion, the crucial therapeutic approach of MGD is to relieve the obstruction in the meibomian glands for improvement of tear stability 1.

Physiotherapy is regarded to be the conventional therapy for removing obstructed meibum in MGD patients, and among various physiotherapies the most commonly applied is warm compress 4. Though warm compress is confirmed to be effective for MGD, manual eyelid massage could be very painful and discomfortable. In addition, warm compress only heat meibomian gland through outer surface of the eyelid, so the temperature is not enough to melt solidified meibum sufficiently and lead to partial and temporary relief of meibomian glands obstruction 5, 6. Thus, these disadvantages of warm compress may frustrate patients and beset ophthalmologists. To overcome the limitations of conventional treatment, any innovative and effective treatment for MGD has certain clinical application prospect. LipiFlow® (TearScience Inc., Morrisville, NC) thermal pulsation, which provides sufficient heat energy from the inner surface of the eyelids accompanied with automated pressure massage from the external eyelids simultaneously, can successfully surmount the limitations of current treatments in dredging the blocked meibomian glands 7. Several randomized controlled trials (RCTs) have demonstrated a single 12-min thermal pulsation treatment can improve tear film stability and relieve related symptoms for MGD patients, however, the underlie mechanism of LipiFlow® has not been explicit verified. According to the latest consensus, inflammation has been emphasized to be the core pathogenesis of MGD and dry eye 8, 9. Therefore, researches focusing on inflammation in MGD are steadily on the increase, but there are rare reports focusing on the variation of chemokine inflammation family in MGD patients.

To address this issue, a prospective interventional study was conducted to investigate the effect of thermal pulsation treatment on meibomian gland function, ocular parameters and tear chemokines compared with the conditional manual eyelid massage. Furthermore, the correlations between MGD-related markers and chemokines were certified to identify the possible mechanism of thermal pulsation treatment. The purpose of the current study aimed to characterize the therapeutic effect of thermal pulsation treatment and its possible mechanism among MGD patients.

## Materials and methods

### Participants

This study was conducted in accordance with the tenets of the Declaration of Helsinki and was approved by the Institutional Review Board (IRB) of the Eye, Ear, Nose, and Throat (EENT) Hospital of Fudan University. The clinical trial was registered in the Chinese Clinical Trial Registry on June 2019. The registration number is ChiCTR-1900023732. Fifty patients who experienced MGD were recruited from the EENT hospital from September 2019 to December 2019. According to consensus of The International Workshop on Meibomian Gland Dysfunction, MGD was confirmed by meibomian gland function examination by the same ophthalmology 1, 10.

The inclusion criteria for MGD based on DEWS II: OSDI score > 12.5 points, FBUT < 10 s; the presence of lid margin abnormalities, orifice abnormalities, and meibum abnormalities 1, 10. These MGD patients aged above 18 years old, who voluntarily participated in the experiment. Subjects with certain ocular diseases (acute ocular inflammation, obvious scar or keratinization in palpebral margin) or receiving physiotherapy for blepharitis (intense pulsed light, baby shampoo, and demodex blepharitis treatments) in the last 3 months may confound the study results; thus, they were excluded from the study. Subjects were also excluded if they had a related ocular surgery, including cataract surgery, trichiasis surgery, lachrymal duct obstruction, or refractive surgery in the past 3 months. After the procedure and potential consequences of the study were explained elaborately, informed consent was obtained from all subjects before the experiment.

At every follow-up, visual acuity, intraocular pressure and anterior segment were observed so as to ensure the safety of treatment. Subsequently, all participants received ocular surface examination including OSDI, meibomian gland expressibility (MGE), meibomian gland quality (MQ), lid margin, meibograde, fluorescein tear film break-up time (FBUT), non-invasive tear break-up time (NIKBUT), corneal fluorescein staining (CFS), Schirmer I test (SIT), tear meniscus height (TMH), lipid layer thickness (LLT) and incomplete blink rate (%).

### MGD related parameters and OSDI

#### Meibomian gland function

Meibomian gland function was evaluated using MGE score, MQ score and lid margin score. The assessments were produced under slitlamp to grade MGE score, MQ score, and lid margin score. Five glands of the middle third of the upper lid were digitally pressed by MG evaluator (MGE‑1000; TearScience), and the MGE was graded as 0-3: grade 0, all five glands expressible; grade 1, 3-4 glands expressible; grade 2, 1-2 glands expressible; and grade 3, no glands expressible 11. Based on the phases of meibum, MQ graded as follows: grade 0, clear; grade 1, cloudy; grade 2, cloudy with granular particulates; and grade 3, thick, like toothpaste-like particulates. Each of the eight glands of the lower eyelid was graded on the scale from 0 to 3. The scores of the eight glands were summarized (range: 0-24) 12. According to the anomalous of the lid margin, lid margin score was graded as 0-4: grade 0 (absent of abnormal); present for the anyone of following parameters recorded as 1: plugged meibomian gland orifices, vascular congestion, irregularity of the lid margin, and partly expressions of the mucocutaneous borderline 13. Combined with upper and lower eyelid margin, the total score ranges from 0-8.

#### Meibograde

Meibographies of the upper and lower eyelids were captured by the Oculus Keratograph 5M (Wetzlar, Germany), and the meibomian gland dropout rate was analyzed qualitatively by ImageJ software (National Institutes of Health, USA). Meibograde of each eyelid was scored based on the meibomian gland dropout rate: 0, meibomian gland area of loss = 0%; 1, dropout rate less than 1/3 of the meibomian gland; 2, dropout rate ranges from 1/3-2/3 of the meibomian gland; and 3, dropout rate was over 2/3 of the meibomian gland. Meibogrades of the upper and lower eyelid were summed to grade as 0-6 for each eye 14.

#### TMH and NIKBUT

TMH and NIKBUT were measured by an OCULUS Keratograph 5M (Wetzlar, Germany) equipped with modified TF-scan software. The procedure was repeated three times following the instructions of OCULUS Keratograph 5M by the same ophthalmology in a dark room. TMH was manual gauged at the central point of the lower lid margin on the images. Then, all participants were required to natural blink twice and then keep their eyes open as much time as possible until the next blink, the duration is defined as NIKBUT.

#### FBUT

A fluorescein strip (Jingming) moistened with preservative‑free saline gently touched the central lower lid margin. After participants blink several times to ensure adequate coating of the complete cornea, then they were required to rapidly open the eyes and this point was recorded as the starting point (time=0 sec). FBUT was defined as the interval between the starting point and the first black spot appearing in the stained team film with a cobalt blue filter and slit lamp microscope. The test was repeated three times and the average FBUT was calculated.

#### OSDI

The OSDI questionnaire, containing a 12‑item questionnaire with a scale of 0‑100, has been designed to rapidly evaluate different ocular discomfort symptoms (soreness, light sensitiveness, blurred vision). The OSDI questionnaire provides a rapid assessment of vision‑related dyspraxia (difficulty reading, driving, operating a computer and watching TV). There is a positive correlation between OSDI scores and the severity of ocular discomfort, with higher scores representing greater ocular discomforts.

#### LLT and incomplete blink rate

LLT and incomplete blink rate were detected non-invasively by the LipiView® instrument (TearScience, Morrisville, NC, United States). All participants were instructed to blink naturally to record a 15 s video of the tear film interference pattern and analyze the LLT, incomplete blink rate (%). The procedure was repeated twice times for each eye.

#### CFS

The steps of corneal staining were similar to that for the assessment of the FBUT. The whole cornea was divided into five zones (central, superior, temporal, nasal and inferior). Corneal epithelial injury was graded on a scale from 0 to 3: 0, no epithelial injury; 1, <30 corneal punctate stains; 2, >30 corneal punctate stains but not fusion; and 3, fusion of corneal staining or ulcer. The total CFS score ranged from 0 to 15.

#### SIT

A sterile dry strip (Jingming®) was inserted into the lateral canthus of the lower eyelid away from the cornea for 5 min. The wetted length of the strip absorbed with tears was recorded as SIT to assess tear secretion. Potential SIT range is from 0 to 30 mm.

### Tear inflammation cytokines

#### Tear sample collection

A disposable 2.2 μL tear collectors (Seinda, Guangdong, China) was applied to obtain tear sample at the lateral canthus before treatment and at 1 month posttreatment. A total amount of 10 μL tear was collected without anesthesia or irritation of the cornea, conjunctiva. Tear samples were transferred into little microtubes immediately and then stored at -80°C for further assays.

#### Assays for tear inflammation cytokines

MILLIPLEX MAP High Sensitivity T Cell Magnetic Bead Panel (Merck EMD Millipore, Billerica, MA, United States) for monocyte chemotactic protein-1 (MCP-1), monokine induced by IFN-γ (MIG), interferon-gamma (IFN-γ), interleukin (IL)-8, and interferon-inducible protein-10 (IP-10) was used according to the manufacturer's instructions. Luminex liquid suspension chip detection was performed using Huaying Biotechnologies (Shanghai, China). Briefly, tear samples were incubated in microbead-embedded 96-well plates overnight at 4°C, and subsequently incubated with detection antibody for 1 h at room temperature at next day. Next, streptavidin-phycoerythrin was added into each well of the plate and incubated for 30 min at room temperature, and the values were detected by a Luminex 200 system (Luminex Corporation, Austin, TX, United States).

### Treatment procedure

In the thermal pulsation group, participants underwent a single 12 min treatment session of LipiFlow® thermal pulsation after local anesthetic as instructed by the manufacturer. A single 12 min automated therapeutic procedure includes an initial heating phase followed by pulsating pressure, which serves to warm and soften glands meibum. Along with the continuous and sufficient heating of meibomian glands, the bladder inflates and deflates in circle so as to massage the eyelids and the meibomian glands thoroughly.

In the control group, participants were instructed to warm the eyelid with spontaneous steam eyelid masks 20 min and do manual lid massage 15 min once daily night. The treatment procedure was performed every night for the entire study duration (12 weeks).

A simple survey focused on tolerance and acceptance with treatment was obtained following treatment. Completed with the entire duration, whether any discomfort (pain, irritation or other) existed during treatment was recorded. Subsequently, the tolerance questionnaire provides a rapid assessment of pain and discomfortable in treatment. The total score of the questionnaire ranges from 1 to 10 points, and higher scores represented more severe discomforts.

### Statistical analyses

Data were analyzed using SPSS v.17.0 software (SPSS inc.) and R-4.1.0 (Copyright (C) 2022 The R Foundation for Statistical Computing). Categorical data were presented as frequency (%). Continuous variables were presented as mean ± standard deviation for normal distribution data or median (25th-75th) for non-normal distribution data. Difference of baseline data between two independent groups were evaluated for statistical significance using the Chi-square test, T test or Wilcoxon test. Repeated-measures ANOVA were conducted to analyze the measures difference between two groups taken at baseline, 1 month, and 3 months after treatment. Paired t test was used to analyze the difference between two time-points. T test was used to analyze the difference of change in various measurements between two groups. The correlations between two measurements with thermal pulsation therapy were performed using the Pearson correlation method. FDR was used to adjust p value for correlations test. P<0.05 was considered statistically significant.

About size of sample calculation, we endeavored to detect a 20% difference in the primary outcome of improved parameters (MGE score and MQ score) between thermal pulsation and control groups according to a previous study 15. Calculated with PASS 11 software, 22 participants in each group were required for 80% power and a two-sided significance level of 5%. Thus, we previously estimated to recruit 25 participants per group to allow for less than 4 cases of lost or withdrawals.

## Results

### Demographic data and baseline clinical characteristics

A total of 25 participants underwent thermal pulsation treatment (8 males and 17 females, aged 36.24 ± 9.82 years), while 25 participants received manual warm compress treatment (10 males and 15 females, aged 34.68 ± 6.02 years). No significant differences in terms of sex (c2=0.103, P = 0.565) and age (P = 0.520) were found between the two groups. There were no differences in the MGD related parameters and OSDI between the thermal pulsation and control groups prior to treatment (all P > 0.05; Table 1). Due to Covid 19 epidemic, there were two subjects in each group were lost during 3 months follow-up.

### MGD related parameters and OSDI

#### Meibomian gland function

**Figure 1:**
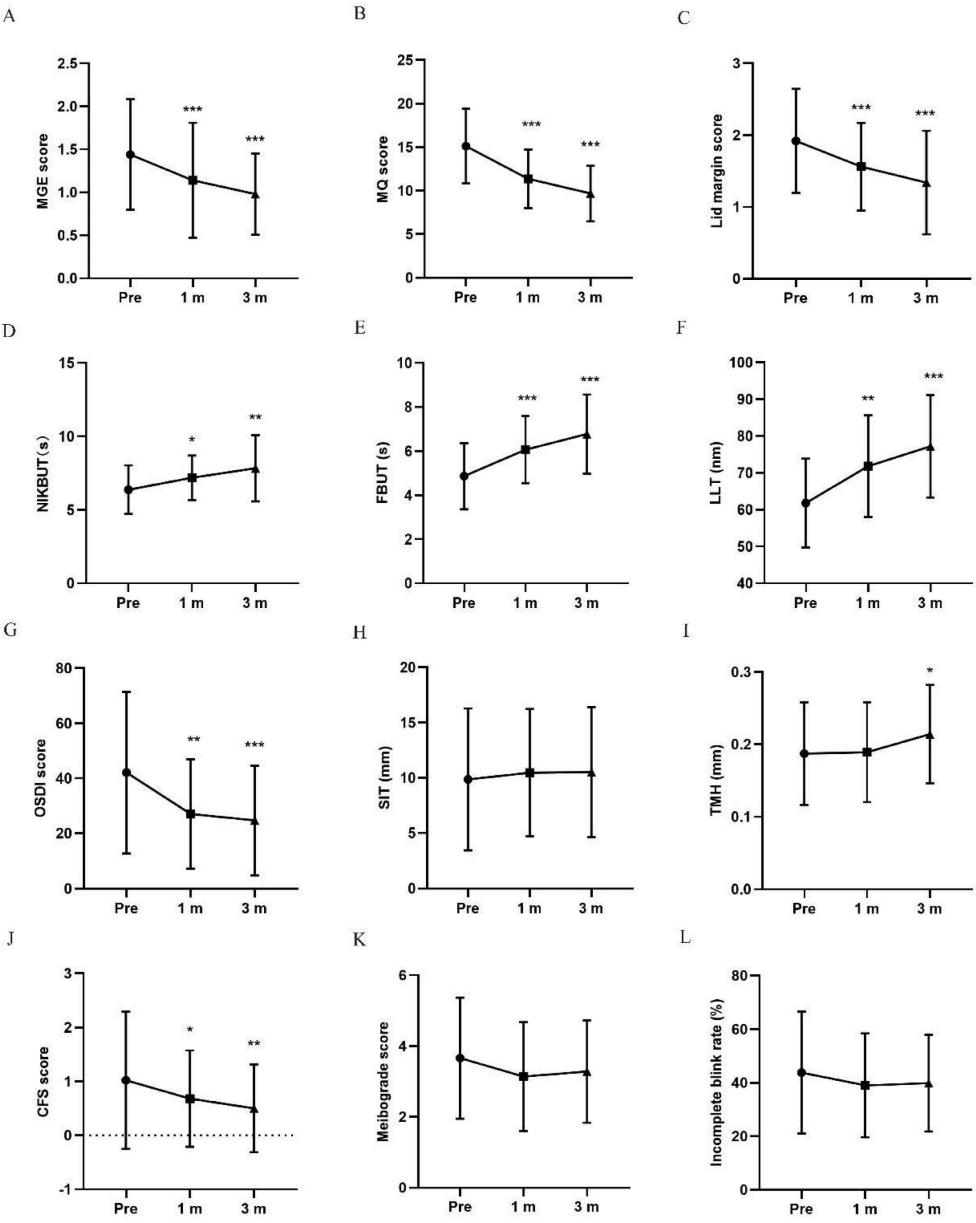
Changes in MGD related parameters and OSDI at 1 and 3 months posttreatment in thermal pulsation group. (A) MGE score. The MGE score significantly decreased in thermal pulsation group at 1 and 3 months posttreatment (***p < 0.001, ***p < 0.001). (B) MQ score. The MQ score significantly decreased in the thermal pulsation group at 1 and 3 months posttreatment (***p < 0.001, ***p < 0.001). (C) Lid margin score. The lid margin score significantly decreased in thermal pulsation group at 1 and 3 months posttreatment (***p < 0.001, ***p < 0.001). (D) NIKBUT. The NIKBUT significantly increased in thermal pulsation group at 1 and 3 months posttreatment (*p = 0.039, **p = 0.007). (E) FBUT. The FBUT significantly increased in thermal pulsation group at 1 and 3 months posttreatment (***p < 0.001, ***p < 0.001). (F) LLT. The LLT significantly increased in thermal pulsation group at 1 and 3 months posttreatment (**p = 0.002, ***p < 0.001). (G) OSDI score. The OSDI score significantly decreased in thermal pulsation group at 1 and 3 months posttreatment (**p = 0.002, ***p < 0.001). (H) SIT. The SIT showed no significant difference in thermal pulsation group at 1 and 3 months posttreatment (p = 1, p = 1). (I) TMH. The TMH showed no significant difference in thermal pulsation group at 1 month posttreatment (p = 0.981), while significant difference in thermal pulsation group at 3 months posttreatment (*p = 0.042). (J) CFS score. The CFS score showed significant difference in thermal pulsation group at 1 and 3 months posttreatment (*p = 0.03, **p = 0.009). (K) Meibograde score. The meibograde score showed no significant difference in thermal pulsation group at 1 and 3 months posttreatment (p = 0.249, p = 0.053). (L) Incomplete blink rate. The incomplete blink rate showed no significant difference in thermal pulsation group at 1 and 3 months posttreatment (p = 0.42, p = 0.708). Paired t test result with thermal pulsation therapy using Bonferroni. Significant differences between pretreatment and posttreatment values. *p < 0.05, **p < 0.01, ***p < 0.001.

The meibomian gland function was comprehensively evaluated by MGE score, MQ score and lid margin score. MGE scores and MQ scores obviously decreased in participants with thermal pulsation at 1 and 3 months posttreatment (***p < 0.001, ***p < 0.001, Figure 1A, B). The lid margin scores gradually decreased at 1 month (***p < 0.001) and 3 months posttreatment in thermal pulsation participants (***p < 0.001; Figure 1C). Although the changes in MGE and MQ score in thermal pulsation group were slight better than warm compress group, no significant difference was found in the improvement in these two parameters at 3 months posttreatment (P > 0.05, Table 2). However, statistics difference was found in the amelioration of lid margin between two groups (P = *0.021, Table 2). Base on above results, thermal pulsation therapy can obvious effectively improve the meibomian gland function for at least 3 months in MGD patients.

#### Tear film stability and LLT

NIKBUT, FBUT, and LLT are three major index of tear film stability. The values of NIKBUT and FBUT were both found significantly extend at 1 and 3 months posttreatment (NIKBUT: *p = 0.039, **p = 0.007; FBUT: ***p < 0.001, ***p < 0.001; Figure 1D, E) in participants with thermal pulsation treatment. Although the extend in NIKBUT and FBUT in thermal pulsation group were bits of longer than warm compress group, no significant difference was found in the extension in NIKBUT and FBUT at 3 months posttreatment (P > 0.05, Table 2).

Participants with thermal pulsation treatment exhibited a thicker LLT at 1 and 3 months posttreatment (**p = 0.002, ***p < 0.001; Figure 1F). Compared with warm compress therapy, thermal pulsation can more effectively thicken the LLT for at least 3 months among MGD patients (**p = 0.008, Tab II).

#### OSDI

The OSDI scores mildly decreased at 1 and 3 months posttreatment in thermal pulsation participants (**p = 0.002, ***p < 0.001; Figure 1G). The OSDI score declined more evident in thermal pulsation group than warm compress group, so a significant difference was found in changes of OSDI score at 3 months posttreatment between two groups (*P = 0.014, Table 2). Based on OSDI values, thermal pulsation therapy can notably alleviate MGD related ocular symptoms for at least 3 months.

### Tear secretion and corneal epithelium injury

Tear secretion was assessed by SIT and TMH. No change was found in the SIT (p = 1, p = 1), TMH at 3 months after thermal pulsation therapy had a slight increase (p = 0.863, *p = 0.042; Figure 1H, I). Compared with warm compress group, there was no significant difference in the improvement of SIT and TMH at 3 months posttreatment (all P > 0.05, Table 2).

Prior to treatment, slight corneal epithelium injury was detected in partial participants of both groups. After treatment, participants accepted thermal pulsation showed less corneal epithelium injury at 1 and 3 months posttreatment (*p = 0.03, **p = 0.009; Figure 1J). The CFS score declined more evident in thermal pulsation group than warm compress group, so a significant difference was found in changes of CFS score at 3 months posttreatment between two groups (*P = 0.027, Table 2).

### Meibograde and incomplete blink rate

**Figure 2:**
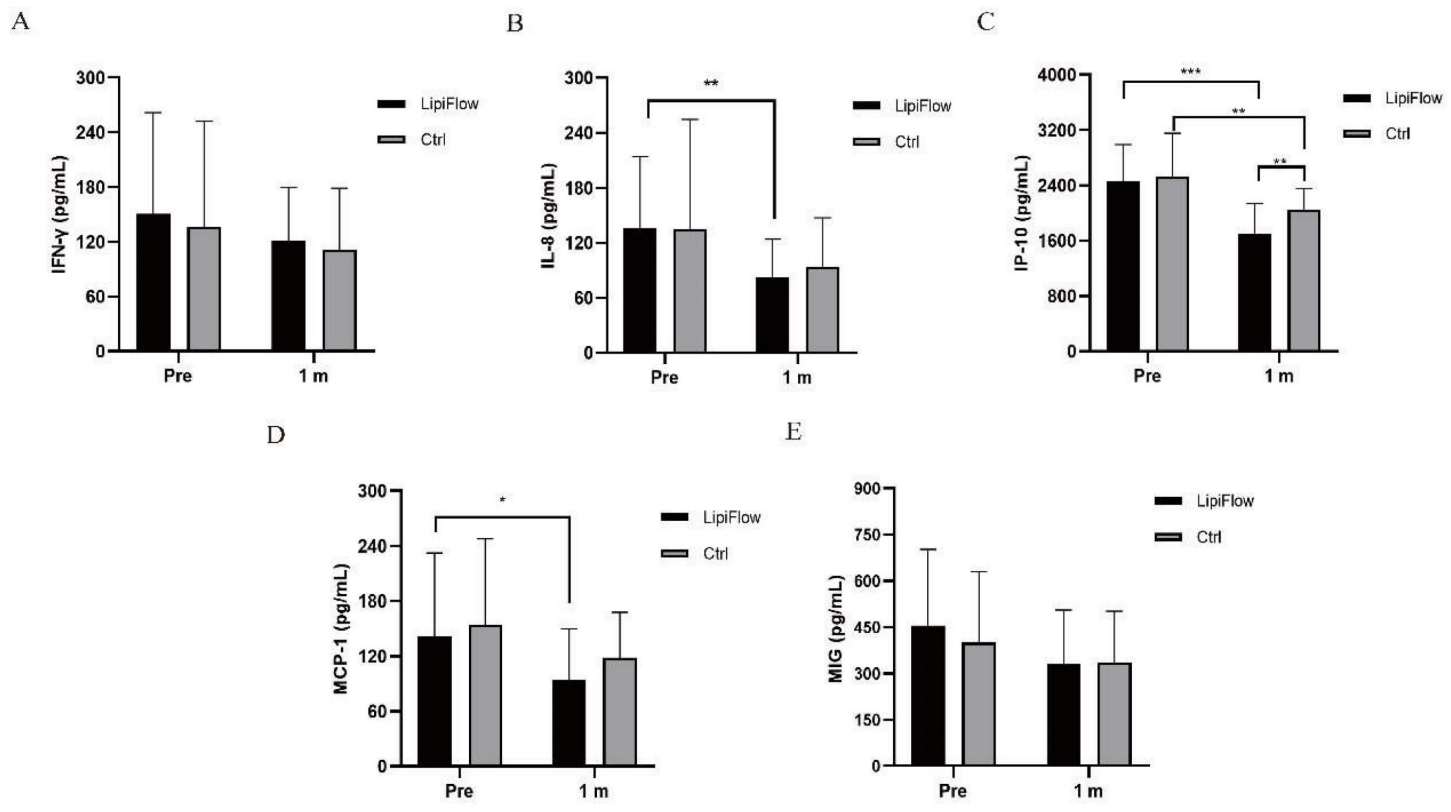
Changes in concentration of tear inflammatory cytokines. (A) No significant difference was found in IFN-γ from baseline and posttreatment levels in subjects in both groups (p > 0.05). (B) The concentration of IL-8 significantly decreased at 1 month in thermal pulsation group (**p = 0.006). (C) The concentration of IP-10 significantly decreased at 1 month in both groups (***p < 0.001, **p = 0.003). (D) The concentration of MCP-1 showed obvious change at 1 month posttreatment (*p = 0.039). (E) No significant difference was found in MIG from baseline and posttreatment levels in subjects in both groups (p > 0.05). Significant difference between the concentration of tear inflammatory cytokines pretreatment and posttreatment. *p < 0.05, **p < 0.01, ***p < 0.001.

Decreasing trends in meibograde score were found in both groups after treatment. However, no significant difference was observed in meibograde at any follow-up time in either participants with thermal pulsation treatment or warm compress treatment (all P > 0.05; Figure 1K, 2K). In incomplete blink rate, no significant changing trend exhibited following thermal pulsation and warm compress groups (all P > 0.05; Figures 1L, 2L). Compared with warm compress group, there was no significant difference in the improvement at 3 months posttreatment (all P > 0.05, Tab II).

### Tear inflammation cytokines

**Figure 3:**
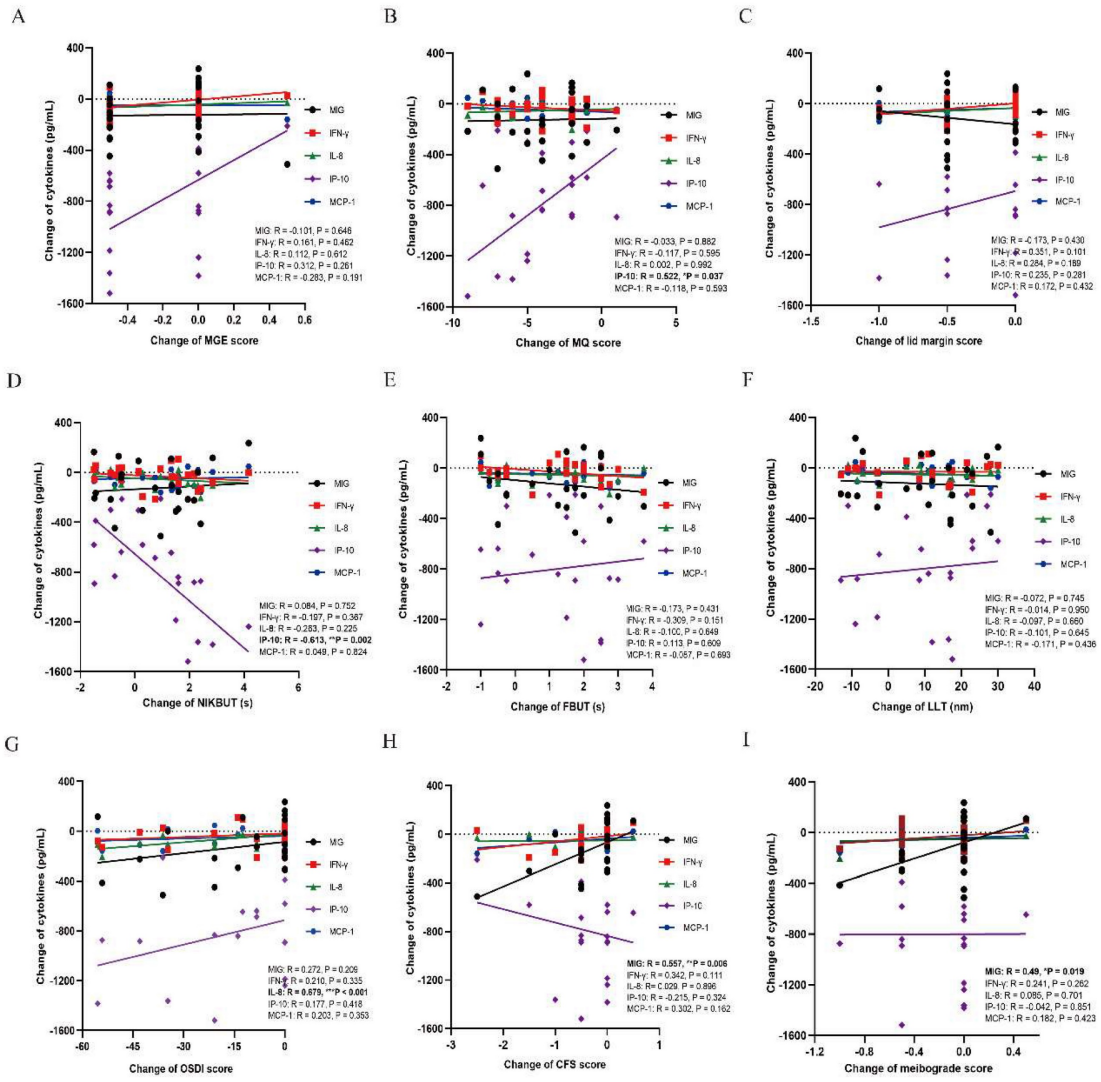
Correlations between MGD related parameters and each inflammatory cytokine. (A) MGE score. No significant correlation is found between MGE and each inflammatory cytokine (all P > 0.05). (B) MQ score. The level of IP-10 is positively correlated with the MQ score (r = 0.522, *p = 0.037). No significant correlation is found between MQ and other inflammatory cytokines (all P > 0.05). (C) Lid margin score. No significant correlation is found between lid margin score and each inflammatory cytokine (all P > 0.05). (D) NIKBUT. The level of IP-10 is negatively correlated with the NIKBUT (r = -0.613, **p = 0.002). No significant correlation is found between NIKBUT and other inflammatory cytokines (all P > 0.05). (E) FBUT. No significant correlation is found between FBUT and each inflammatory cytokine (all P > 0.05). (F) LLT. No significant correlation is found between LLT and each inflammatory cytokine (all P > 0.05). (G) OSDI score. The level of IL-8 is positively correlated with the OSDI score (r = 0.679, ***p < 0.001). No significant correlation is found between OSDI score and other inflammatory cytokines (all P > 0.05). (H) CFS score. The level of MIG is positively correlated with the CFS score (r = 0.557, **p = 0.006). No significant correlation is found between CFS score and other inflammatory cytokines (all P > 0.05). (I) Meibograde score. The level of MIG is positively correlated with the meibograde score (r = 0.49, *p = 0.019). Significant differences between the correlations with tear inflammatory cytokines and MGD related parameters. The bold values mean significant results. *p < 0.05, **p < 0.01, ***p < 0.001.

Five inflammatory cytokines (MIG, IFN-γ, IL-8, IP-10, and MCP-1) were examined and analyzed compared between participants in warm compress and thermal pulsation groups before the treatment, and after 1 month treatment. Based on inflammatory cytokines results, no significant differences of the baseline levels were found between these five inflammatory cytokines in two groups subjects (all p > 0.05; Figure 2A-E, Table 3). Decreasing trends of these five inflammatory cytokines were identified at 1 month posttreatment in participants (Figure 2A-E). In thermal pulsation group, the levels of IL-8, IP-10 and MCP-1 declined obviously than the baseline levels at 1 month posttreatment (**p = 0.006, ***p < 0.001, and *p = 0.039; Figure 2B-D, Table 3). No significant differences of IFN-γ and MIG were found between pretreatment and posttreatment in thermal pulsation group (Figure 3A, E, Table 3). In participants with warm compress, only the level of IP-10 significantly declined at 1 month posttreatment (**p = 0.003; Figure 2C, Table 3). Compared with warm compress group, there was more decline in IP-10 and MIG at 1 month posttreatment (IP-10: *P = 0.021, MIG: *P = 0.039, Table 3).

### The correlations between tear inflammatory cytokines and MGD related parameters

There were decrease trends in all inflammatory cytokines after either thermal pulsation or warm compress treatment. In MGD related parameters, the decrease of IP-10 was found positively correlated with the improvement of meibomian function (MQ: r = 0.522, *p = 0.037; Figure 3B), while decline of IP-10 was negatively correlated with worse tear stability (NIKBUT: r = -0.613, **p = 0.002; Figure 3D). Moreover, the decrease of MIG was positively correlated with less corneal epithelium injury (CFS: r = 0.557, **p = 0.006; Figure 3H) and meibograde (meibograde: r = 0.49, *p = 0.019; Figure 3I). In terms of subjective symptoms, only IL-8 was found positively correlated with less ocular symptoms (OSDI: r = 0.679, ***p < 0.001; Figure 3G). However, the levels of MCP-1 and IFN-γ were not found to be significantly correlated with any of the MGD related markers (all P > 0.05; Figure 3A-H). The level of IP-10 and MIG was identified to be affected MGD related parameters and tear film stability (Figure 3B, D, H, I), while the symptoms was only found to be correlated with IL-8 level (Figure 3G).

### Safety index and Tolerance

No visual loss and IOP elevated above 21mmHg were recorded during all treatment in two groups. Only 1 of the 25 participants accepted the thermal pulsation treatment reported slightly irritation, other participants reported no pain and discomfort during application. However, 10 of 25 participants reported mild to severe pain and discomfortable in warm compress group (*** P < 0.001; Table 4). The average score of tolerance questionnaire was 3.88 ± 1.740 in control group, while that of thermal pulsation was 0.20 ± 0.408 (*** P < 0.001, Table 4). The thermal pulsation treatment caused less sore and accompanied with more comfortable than manual eyelid massage. Hence, the relaxed treatment process of the thermal pulsation treatment guaranteed better compliance among MGD patients.

## Discussion

In the current study, the effectiveness, safety, and sustainability of a single thermal pulsation treatment was verified compared with at-home warm compress therapy over 3 months period. Overall, both the thermal pulsation and warm compress groups showed obvious improvements in MGD related parameters and subjective symptoms. In contrast with control group, the thermal pulsation group exhibited better outcomes in several parameters (MGE, MQ, and CFS), and they were statistically significant. Measured at 3 months post thermal pulsation treatment, the mean meibomian gland function score had decreased about 30-40% from baseline and the mean OSDI symptom score declined by about 50%. The result was consistent with the study of David Badawi 16 that thermal pulsation treatment demonstrated a significant improvement in TBUT, meibomian gland scores as well as corneal and conjunctival staining scores than the subjects in the WC group for 6 months. Similar results were reported by Blackie et al. 17, a single VTP treatment can provide significantly greater mean improvement in meibomian gland function and dry eye symptoms in dry eye patients with good safe.

LipiFlow® is an automated MGD therapeutic device, which offers an effective treatment for meibomian glands obstruction. An explanation for the good efficacy of thermal pulsation is that LipiFlow® can sufficiently squeeze the eyelids to clear out the obstruction in the meibomian glands after the meibum fully melting. The device delivers constant 42℃ heat directly over the meibomian glands of the upper and lower inner eyelids while varying degree of pulsatile pressure simultaneously evacuates the obstruction meibum. This temperature is the optimal temperature intended to effectively melting of obstructed meibum, while posing no risk to ocular surface at the same time 18. Considering that the meibomian glands are located on the inner eyelid, to achieve and maintain a therapeutic heat temperature on inner eyelid is an important prerequisite for subsequent meibomian gland dredging and treatment. Moreover, particularly notable was the better tolerance of the thermal pulsation treatment which can guarantee great compliance during long treatment duration. Compared to conventional treatments for MGD patients with apparent ocular pain and discomforts, a single thermal pulsation can overcome the limitations of conventional methods to obtain satisfactory curative effect and maintain for at least 3 months.

In the DEWS II report, the etiology of MGD was described to be terminal duct obstruction with or without qualitative or quantitative changes in the glandular lipid secretion, impairing ocular surface homeostasis and lead to tear hyperosmolarity and apparent inflammation 8, 19. In turn, tear hyperosmolarity promotes the induction of inflammatory cytokines, which then causes tear film instability, impaired structure and function of meibomian gland, goblet cell loss, and less mucin production - all of which further aggravate the hyperosmolarity of tear 20, 21. Based on the above mechanism, an uninterrupted vicious circle is formed between MGD and inflammation. The previous report 22 showed that increased levels of various inflammatory cytokines (IL-6, IL-8, TNF-α, and IFN-γ) were found in MGD patients, and these inflammatory cytokines were also associated with meibomian gland function and tear stability. Another study 23 investigated the tear inflammation cytokines between normal subjects and MGD patients, and it was observed that inflammation cytokines (such as TNF-α, IL-1β, IL-6, IL-8, IL-12p70 and IFN-γ) significant elevated in the tear of MGD patients. These results verified that worse meibomian gland function was closely associated with a higher level of inflammation cytokines. Thus, an inseparable relationship between the abnormalities of the meibomian glands is in association with ocular surface inflammation 8.

In our study, meibomian gland function and tear film stability improved accompanied with the decrease of chemokines in MGD patients. The decrease in IP-10 was positively correlated with improvement of MQ, while it was negatively correlated with tear instability. Considering the correlation between MGD and inflammation, thermal pulsation treatment can effectively evacuate the obstruction meibum in meibomian gland to reduce the concentration of inflammatory factors in ocular surface and improve the stability of tear film. IP-10, known as CXCL-10, is an ELR‑negative CXC chemokine induced by IFN-γ or other stimuli during infection or inflammation in several immune cell 24. Combined the robust immunomodulatory effect of IP-10/CXCL-10 25 with the highly expression of IP-10/CXCL-10 in MGD patients, we speculated that IP-10/CXCL-10 plays a critical role in the pathogenesis of MGD. According to relevant studies CXC chemokines are potent inhibitors of oxidative stress 26, and inhibition of IP-10/CXCL-10 can definitely guarantee better outcomes in acute ischemic stroke patients by abating obvious oxidative stress 27. The anti-oxidative treatment effect of IP10/CXCL-10 have been confirmed in the treatment of various diseases 28, 29. Although the etiology of MGD has not yet been fully illustrated, the involvement of the immune system and oxidative stress could be considered as common accepted theories among various mechanisms could explain the progression of MGD pathology. In the Cu, Zn-Superoxide Dismutase-1 (SOD-1) knockout mice 30, larger lipid droplets and more apoptosis of meibomian gland epithelial cells were found compared with wild type mice, so excessive oxidative response is relative with the worse meibomian gland function. In addition to animal experiments, several clinical trials also confirmed the role of oxidation response in maintaining the stability of tear film. Louis Tong 31 once reported that the level of protein S100A8 was significantly correlated to grittiness and protein S100A9 was correlated to symptoms of redness and transient blurring possibly by triggering the excessive oxidative response in MGD patients. MIG, a chemokine monokine induced by IFN-γ/CXCL-9, can stimulate T lymphocyte proliferation and effector cytokine production. MIG, as a member of CXCL family chemokines, is also found to be closely related to corneal injury and meibomian gland function in our study. In the light of the tight relationship between oxidative stress to inflammation, the therapy targeted on preventing oxidative stress can reduce ocular surface damage or improved lubricity and wound healing in MGD 32, 33. Hence, we speculated that the decline of CXCL family cytokines after thermal pulsation treatment may potentially achieve the therapeutic effect on MGD probably by attenuating oxidative stress response in MGD. Based on the transitional inflammation and oxidative stress response in MGD, systemic and topical anti-inflammation treatment should be accepted in regular strategies to maintain the normal function of meibomian glands, and especially novel agent targeted on CXCL family cytokines may have great prospects in the treatment of MGD.

Furthermore, positive correlations were found in our study between IL-8 and dry eye symptoms, which agreed with the correlations reported by Zhao et al. 14 and Zhang et al. 34. IL-8, also named as CXCL-8, is the most well-known molecule in the chemokine family which has great attractive chemotactic effects to neutrophils, lymphocytes and basophils 35. In contrast with normal people, the chemokine IL-8/CXCL-8 was found significantly elevated in all kinds of dry eye patients tear, therefore, IL-8/CXCL-8 may be located in the critical position in the pathogenesis of dry eyes and strongly affects ocular discomfort symptoms 22, 35. The excessive oxidative response may also lead to cytotoxicity and apoptosis on the ocular surface, meibomian gland and lacrimal gland tissues through the amplification cascade expression of IL-8/CXCL-8 36, 37. Thus, inhibition of IL-8 can drastically deter the inflammation and significantly improve the MGD-related dry eye symptoms. In view of MGD related subjective symptoms and objective signs closely related with IL-8/CXCL-8 and IP-10/CXLC-10 respectively, therefore, we supposed that chemokines, especially IL-8/CXCL-8 and IP-10/CXLC-10, can be detected as sensitive biomarkers in pre-evaluation of efficacy of MGD treatment.

Due to the limitation of the small number of participants and short follow-up duration in our study, the efficacy of thermal pulsation treatment for MGD patients requires further verification in a larger sample with a longer follow-up period. Furthermore, inadequate analysis of inflammatory cytokines was also a deficiency in the present study. In addition, no evaluation was conducted on the effects of other physiotherapy MGD treatments, such as intense pulsed light (IPL). Thus, further studies should be conducted with a larger number of participants examined with more analysis of inflammatory cytokines and more kinds of treatment, so as to explicit illustrate the therapeutic effect of thermal pulsation in MGD patients.

This study focused on exploring the therapeutic effect of thermal pulsation treatment on MGD patients and its underlying mechanism. A single 12 min thermal pulsation treatment can effectively alleviate MGD-related parameters and improve tear film stability for at least 3 months. Moreover, thermal pulsation treatment presented better ocular tolerance, and it could be more comfortable than the manual eyelid massage. We found CXCL cytokines are closely related with MGD related parameters and tear film instability in thermal pulsation treatment. Thus, we speculated that the therapeutic effect of thermal pulsation treatment might be related with the decline of tear inflammatory cytokines, especially chemokine family. Considering that MGD is a chronic disease, it is beneficial for MGD patients undergoing the thermal pulsation treatment to achieve curative effect for at least 3 months and avoid obvious pain and discomforts accompanied with frequent manual eyelid massage.

## Figures and Tables

**Table 1:** Comparison of demographic data and baseline clinical characteristics of between the thermal pulsation and warm compress groups (Mean ± SD).

	Thermal pulsation	Warm compress	P Value
Age (years)	36.24 ± 9.82	34.68 ± 6.02	0.502
F/M	17/8	15/10	0.565
OSDI score	42.06±29.29	40.88±24.16	0.877
MGE score	1.44±0.64	1.64±0.63	0.120
MQ score	15.12±4.298	14.60±4.106	0.538
Meibograde	3.66±1.71	3.50±1.68	0.638
NIKBUT (s)	6.37±1.65	6.72±1.72	0.297
FBUT(s)	4.29±1.50	5.03±1.15	0.526
LLT (nm)	61.78±12.05	65.20±12.28	0.163
SIT (mm/5 minutes)	9.86±6.42	10.72±6.62	0.551
TMH (mm)	0.187±0.07	0.183±0.08	0.776
CFS score	1.02±1.27	1.04±0.95	0.929
Lid margin score	1.92±0.72	1.72±0.61	0.138
Incomplete blink rate (%)	43.80±22.76	44.40±25.81	0.902

MGE, Meibomian gland expressibility; MQ, Meibomian gland quality; NIKBUT, Non-invasive keratograph tear film break-up time; FBUT, Fluorescein break-up time; LLT, Lipid layer thickness; OSDI, Ocular Surface Disease Index; SIT, Schirmer TMH, Tear meniscus height; CFS, Fluorescein staining score. Age, F/M and OSDI (n=25), other parameters (n=50). Significant differences between thermal pulsation and warm compress values. *p < 0.05, **p < 0.01, ***p < 0.001.

**Table 2:** Comparison of changes in all parameters between the thermal pulsation group and warm compress group at 3 months posttreatment (Mean ± SD).

	Thermal pulsation	Warm compress	P Value
OSDI	-17.38±20.27	-8.82±13.46	*0.014
MGE score	-0.46±0.58	-0.40±0.49	0.579
MQ score	-5.44±3.23	-3.28±3.37	0.267
Meibograde	-0.38±1.28	-0.46±1.05	0.361
NIKBUT (s)	1.46±2.18	1.07±2.33	0.397
FBUT(s)	1.91±0.87	1.49±1.71	0.245
LLT (nm)	15.42±14.01	8.64±10.98	**0.008
SIT (mm/5 minutes)	0.66±6.99	1.04±6.90	0.785
TMH (mm)	0.027±0.06	0.008±0.06	0.093
CFS score	-0.52±0.84	-0.18±0.66	*0.027
Lid margin score	-0.58±0.70	-0.30±0.46	*0.021
Incomplete blink rate (%)	-4.00±18.29	-1.00±16.19	0.387

MGE, Meibomian gland expressibility; MQ, Meibomian gland quality; NIKBUT, Non-invasive keratograph tear film break-up time; FBUT, Fluorescein break-up time; LLT, Lipid layer thickness; SIT, Schirmer TMH, Tear meniscus height; CFS, Fluorescein staining score. OSDI (n=25), other parameters (n=50). Significant differences between thermal pulsation and warm compress values. The bold values mean significant results. *p < 0.05, **p < 0.01, ***p < 0.001.

**Table 3:** Comparison of changes in concentration of tear inflammatory cytokines between the thermal pulsation group and warm compress group at 1 months posttreatment (Mean ± SD).

	Thermal pulsation	Warm compress	P Value
IFN-γ	-30.37±83.36	-25.46±76.22	0.836
IL-8	-61.95±52.39	-32.49±69.22	0.112
IP-10	-808.84±468.05	-474.38±481.64	*0.021
MCP-1	-47.39±84.82	-36.01±82.71	0.606
MIG	-178.29±184.68	-63.87±180.91	*0.039

IFN-γ, interferon-gamma; IL-8, interleukin-8; IP-10, interferon-inducible protein-10; MCP-1, monocyte chemotactic protein-1; MIG, monokine induced by IFN-γ. Significant differences between thermal pulsation and warm compress values. The bold values mean significant results. *p < 0.05, **p < 0.01, ***p < 0.001.

**Table 4:** Comparison of tolerance score between the thermal pulsation and warm compress treatment groups (Mean ± SD).

	Thermal pulsation (n=25)	Warm compress (n = 25)	P Value
Uncomfortable rate	1/25	10/25	*** < 0.001
Tolerance score	0.20 ± 0.408	3.88 ± 1.740	*** < 0.001

Significant differences between the thermal pulsation and warm compress groups. The bold values mean significant results. *p < 0.05, ** p < 0.01, *** p < 0.001.

## Abbreviations

CFS: corneal fluorescein staining
IPL: intense pulsed light
MCP-1: monocyte chemotactic protein-1
MGD: meibomian gland dysfunction
MGE: meibomian gland expressibility
MIG: monokine induced by IFN-γ
MQ: meibomian gland quality
OSDI: Ocular Surface Disease Index
TMH: tear meniscus height
NIKBUT: non-invasive tear break-up time
FBUT: fluorescein tear film break-up time
FL: fluorescein staining
IFN-γ: interferon-gamma
IL-8: interleukin-8
IP-10: interferon-inducible protein-10
LLT: lipid layer thickness
SIT: Schirmer I test
TMH: tear meniscus height
